# Impact of the COVID-19 Pandemic on Outpatient Visit-Based Frequency Trends of Herpes Zoster Ophthalmicus

**DOI:** 10.7759/cureus.109260

**Published:** 2026-05-20

**Authors:** Yuya Furukawa, Mao Tanabe, Hitoshi Tabuchi, Fumi Gomi, Atsuki Fukushima

**Affiliations:** 1 Ophthalmology, Tsukazaki Hospital, Himeji, JPN; 2 Ophthalmology and Visual Science, Nagoya City University Graduate School of Medical Sciences, Nagoya, JPN; 3 Ophthalmology, Hyogo Medical University, Nishinomiya, JPN

**Keywords:** covid-19, herpes zoster ophthalmicus (hzo), hzo, ophthalmology, uveitis

## Abstract

Herpes zoster ophthalmicus (HZO) is a vision-threatening disease, yet the long-term impact of the COVID-19 pandemic on its relative frequency and ocular complication profile remains insufficiently understood. This retrospective observational study aimed to quantitatively evaluate the pandemic's influence on the long-term relative frequency trends of HZO and its ocular complications. We analyzed 15 years of clinical data from January 2011 to June 2025 at a single institution in Japan. Interrupted time series analysis was employed to distinguish immediate level changes from long-term trend changes in disease relative frequency following the pandemic's onset in early 2020. Comparing the pre-pandemic and post-pandemic cohorts, the mean age of patients with HZO increased significantly by approximately eight years, and the proportion of cases complicated by uveitis increased from 22.7% to 41.6%. While the time series analysis indicated no immediate surge in the relative frequency of HZO at the pandemic's start, it revealed a highly significant acceleration in the long-term increasing trend of HZO cases. Furthermore, the frequency trend of herpetic uveitis exhibited a similar and statistically significant upward shift during the post-pandemic period. These findings suggest that the pandemic period was associated with a transient fluctuation in healthcare utilization, but it was also associated with a sustained acceleration in the relative frequency of HZO and a notably higher frequency of HZO-associated uveitis. The pronounced increase in cases among older adults underscores the critical importance of proactive herpes zoster vaccination and enhanced ophthalmic triage to prevent permanent vision loss in the post-pandemic era.

## Introduction

Herpes zoster (HZ), caused by reactivation of the varicella-zoster virus (VZV), is primarily a disease of older adults due to age-related decline in cell-mediated immunity, with incidence typically rising after age 50. Among its forms, herpes zoster ophthalmicus (HZO), which occurs in the ophthalmic branch (first division) of the trigeminal nerve, is a condition that can lead to serious vision-threatening complications such as keratitis and uveitis. Since the start of the COVID-19 pandemic in early 2020, it has been speculated that either direct immunological effects of the SARS-CoV-2 infection or pandemic-related lifestyle changes and stress might have influenced the outpatient visit-based frequency trends of HZ and HZO.

However, reports on the pandemic’s impact on the outpatient visit-based frequency trends of HZ and HZO have been conflicting. A study in Brazil using public health data reported that the number of HZ diagnoses increased by approximately 35% in the early phase of the pandemic [1]. In contrast, Snyder et al. in the United States and Sakamoto et al. in Japan reported that overall HZ clinic visits actually decreased or remained flat, likely due to patients avoiding medical visits (“medical distancing”) [2,3]. Notably, even as overall HZ cases declined, one report (Snyder et al.) observed a specific increase in HZO cases during the pandemic [2]. This suggests that HZO may have distinct triggers or risk factors compared to other forms of HZ, though it remains unclear whether the observed increase in HZO was merely transient.

Moreover, many existing studies analyzed relatively short periods (from the pandemic’s beginning through 2020-2021) [4,5], so knowledge of the longer-term impact of the prolonged pandemic is limited. In particular, very few studies on HZO and its ocular complications have examined that not only immediate case number changes (level changes) but also the longer-term relative frequency trends of HZ and HZO (slopes) were altered by the pandemic. If the increasing outpatient visit-based frequency trends of HZO have accelerated since the onset of the pandemic, this would have important implications for future healthcare demand and herpes zoster vaccination strategies.

Therefore, in this study, we primarily aimed to quantitatively evaluate the impact of the COVID-19 pandemic on the outpatient visit-based frequency trends of HZO from a long-term perspective. The secondary objective was to assess whether the outpatient visit-based frequency trends of major ocular complications, particularly herpetic uveitis, changed after the onset of the pandemic. We analyzed 15 years of clinical data (January 2011 - June 2025) from the ophthalmology department of a single institution in Japan, and applied an interrupted time series (ITS) analysis to distinguish short-term level changes from long-term trend (slope) changes following the introduction of the pandemic.

## Materials and methods

We conducted a retrospective observational study at Tsukazaki Hospital’s Ophthalmology outpatient department. The study period spanned several years before and after the COVID-19 pandemic (from January 1, 2011 to June 30, 2025). For each half-year interval in this period, we evaluated long-term trends in the outpatient visit-based frequency of HZO, defined as the proportion of new HZO cases among all ophthalmology outpatient visits. New HZO cases were identified based on ICD coding or clinical diagnosis records in electronic medical charts. Specifically, HZO was diagnosed clinically based on the presence of a vesicular rash in the V1 distribution with compatible ocular or periocular findings [6,7]. Virological confirmation by PCR was not routinely performed during the study period. Cases without a typical vesicular rash were not included unless the clinical diagnosis of HZO was clearly documented by an ophthalmologist. Ambiguous cases were reviewed using the medical records and excluded when the diagnosis could not be confirmed. We also recorded the presence of ocular complications, such as uveitis, keratitis, ocular motility disorder, and optic neuritis, in each HZO case. Uveitis cases were identified based on chart-documented clinical diagnosis and interpreted with reference to established classification criteria for varicella zoster virus anterior uveitis [8]. For each type of complication, we similarly calculated its relative frequency per half-year as the number of HZO patients with that complication divided by the total number of outpatients.

Continuous variables were summarized as mean ± standard deviation and compared between the pre-COVID (2011-2019) and post-COVID (2020-2025) HZO cohorts using Welch’s t-test. For binary variables with sufficient expected cell counts (sex, any ocular complication, keratitis, and uveitis), proportions were compared using the Yates-corrected chi-square test. For sparse binary outcomes (ocular motility disorder and optic neuritis), Fisher’s exact test was used. For descriptive between-group comparisons, the effect estimate is reported as the mean difference (continuous variables), risk difference (binary variables analyzed by chi-square), or odds ratio (binary variables analyzed by Fisher’s exact test), each with a 95% confidence interval.

Half-year proportions were analyzed using interrupted time-series (ITS) segmented ordinary least squares (OLS) regression. The primary denominator was the total number of ophthalmology outpatient visits in each half-year. The relative frequency of HZO was defined as the number of new HZO cases divided by total outpatient visits, and the relative frequency of ocular complications was defined as the number of HZO cases with the corresponding complication divided by total outpatient visits. Because these outcomes were small proportions with varying denominators, we applied an arcsine square-root transformation before fitting the segmented OLS models. This approach was chosen as a variance-stabilizing transformation for proportions and allowed direct estimation of pre-pandemic trend, immediate level change, and post-pandemic slope change on a common transformed scale. The segmented model included a continuous time counter, a post-pandemic indicator beginning in the first half of 2020, and a post-pandemic time-slope term. The immediate level change (β2) and the post-pandemic slope change (β3) were reported with Wald 95% confidence intervals, Wald z statistics, and two-sided p-values. In this model, β2 represents the immediate change in HZO frequency at the onset of the pandemic, whereas β3 represents the change in the long-term trend after the pandemic began. Cumulative excess cases during the post-pandemic period were calculated as the sum of the difference between the fitted post-pandemic trajectory and the counterfactual trajectory predicted under no pandemic, multiplied by the observed half-year outpatient denominator. Confidence intervals for cumulative excess cases were obtained by parametric bootstrap of the coefficient covariance matrix.

To address time-series model assumptions, residual autocorrelation was assessed using the Durbin-Watson statistic and Ljung-Box tests at lags 1 and 2. Residual stationarity was additionally explored using the augmented Dickey-Fuller test, although stationarity of the raw outcome series was not required because the segmented regression explicitly modeled deterministic changes in level and slope. Potential half-year seasonality was assessed by adding a half-year indicator (H2 vs H1) to the segmented model. Because viral diseases can exhibit seasonal patterns, this season-adjusted model was treated as a prespecified sensitivity analysis.

To evaluate whether the choice of arcsine-transformed OLS materially affected the conclusions, we performed sensitivity analyses using binomial-logit regression and Poisson rate regression with log (total outpatient visits) as an offset. We also fitted an absolute-count Poisson model without an outpatient-visit denominator to evaluate the possibility that changes in healthcare utilization during the pandemic affected the proportion-based results. These sensitivity analyses were interpreted as supportive rather than primary because the study included only 29 half-year periods and several outcomes were sparse.

All analyses were conducted using Python version 3.13.5 (Python Software Foundation, Beaverton, OR, USA). Data processing was performed with pandas 2.2.3 (The pandas Development Team, via NumFOCUS, Inc., Austin, TX, USA) and NumPy 2.3.5 (NumPy Developers, via NumFOCUS, Inc., Austin, TX, USA). Statistical tests were performed using SciPy 1.17.0 (SciPy Developers, via NumFOCUS, Inc., Austin, TX, USA), and interrupted time series models, generalized linear models, and residual diagnostics were performed using statsmodels 0.14.6 (statsmodels Development Team, open-source project maintained on GitHub; globally distributed). Figures were generated using Matplotlib 3.10.8 (Matplotlib Development Team, via NumFOCUS, Inc., Austin, TX, USA).

This retrospective study was approved by the institutional ethics committee, and the requirement for informed consent was waived because of the retrospective nature of the study.

## Results

Patient background changes are summarized in Table 1, and absolute numbers of HZO cases and total outpatient visits for each half-year interval are provided in Table 2. The mean age increased from 57.1 ± 18.3 years in the pre-pandemic cohort to 65.2 ± 18.3 years in the post-pandemic cohort, corresponding to a mean difference of 8.1 years (95% CI 2.4 to 13.7; Welch’s t = 2.82, p = 0.005). Uveitis was more frequent after the pandemic (22.7% vs 41.6%), with an absolute risk difference of 18.9% (95% CI 5.0 to 32.9; Yates-corrected χ² = 5.76, p = 0.016). Any ocular complication also tended to be more frequent post-pandemic (37.3% vs 52.8%), although this did not reach conventional statistical significance (risk difference 15.5%, 95% CI 0.4% to 30.6%; Yates-corrected χ² = 3.33, p = 0.068). Ocular motility disorder remained rare, with one case before and seven cases after the pandemic (odds ratio 6.32, 95% CI 0.76 to 52.56; Fisher’s exact p = 0.072).

**Table 1:** Comparison of patient characteristics before and after the COVID-19 pandemic. Continuous variables are shown as mean ± standard deviation, and categorical variables as number of cases (percentage). In the post-pandemic HZO patient group (Post-COVID: 2020–2025), the mean age was significantly higher (p = 0.005), and the rate of uveitis complication was also significantly higher (p = 0.016) compared to the pre-pandemic group (Pre-COVID: 2011–2019).

Characteristic	Pre-COVID (n=75)	Post-COVID (n=89)	Effect estimate (95% CI)	Test statistic (test)	P-value
Age (years), mean ± SD	57.1 ± 18.3	65.2 ± 18.3	Mean diff = 8.1 (95% CI 2.4 to 13.7)	t = 2.82 (Welch’s t-test)	0.005
Male sex, No. (%)	34 (45.3%)	46 (51.7%)	Risk diff = 6.4% (95% CI -9.0 to 21.7)	χ² = 0.43 (Yates-corrected χ²)	0.513
Female sex, No. (%)	41 (54.7%)	43 (48.3%)	Risk diff = -6.4% (95% CI -21.7 to 9.0)	χ² = 0.43 (Yates-corrected χ²)	0.513
Any ocular complication, No. (%)	28 (37.3%)	47 (52.8%)	Risk diff = 15.5% (95% CI 0.4 to 30.6)	χ² = 3.33 (Yates-corrected χ²)	0.068
Keratitis, No. (%)	24 (32.0%)	32 (36.0%)	Risk diff = 4.0% (95% CI -10.6 to 18.5)	χ² = 0.13 (Yates-corrected χ²)	0.714
Uveitis, No. (%)	17 (22.7%)	37 (41.6%)	Risk diff = 18.9% (95% CI 5.0 to 32.9)	χ² = 5.76 (Yates-corrected χ²)	0.016
Ocular motility disorder, No. (%)	1 (1.3%)	7 (7.9%)	OR = 6.32 (95% CI 0.76 to 52.56)	OR = 6.32 (Fisher’s exact test)	0.072
Optic neuritis, No. (%)	0 (0.0%)	2 (2.2%)	OR = 3.45 (95% CI 0.15 to 77.65)	OR = 3.45 (Fisher’s exact test)	0.501

**Table 2:** Total outpatient visits, HZO counts, proportions, and ocular complication counts by half-year

YearHalf	All_patients	HZO_cases	HZO_prop_per_10000	Any ocular complication	Keratitis	Uveitis	Ocular motility disorder	Optic neuritis
2011-H1	6809	3	4.405933	0	0	0	0	0
2011-H2	7478	3	4.011768	0	0	0	0	0
2012-H1	8286	2	2.41371	0	0	0	0	0
2012-H2	8956	3	3.34971	1	1	1	0	0
2013-H1	9936	5	5.032206	4	2	2	0	0
2013-H2	10866	6	5.521811	3	3	2	0	0
2014-H1	11454	4	3.49223	1	0	1	0	0
2014-H2	12410	2	1.611604	1	1	1	0	0
2015-H1	12999	2	1.53858	1	1	0	0	0
2015-H2	13979	6	4.292153	2	1	2	0	0
2016-H1	14182	6	4.230715	2	2	2	0	0
2016-H2	14726	4	2.716284	3	3	2	1	0
2017-H1	15317	5	3.264347	2	2	2	0	0
2017-H2	16276	6	3.686409	1	1	0	0	0
2018-H1	16929	4	2.362809	2	2	1	0	0
2018-H2	17436	8	4.588208	3	3	1	0	0
2019-H1	17314	5	2.887836	2	2	0	0	0
2019-H2	17489	1	0.571788	0	0	0	0	0
2020-H1	15810	5	3.162555	5	3	3	2	1
2020-H2	16673	4	2.399088	1	1	1	0	0
2021-H1	14871	8	5.379598	3	3	2	0	0
2021-H2	16870	6	3.556609	1	1	1	0	0
2022-H1	16366	6	3.666137	4	1	3	0	0
2022-H2	15770	9	5.707039	4	3	4	1	0
2023-H1	14939	12	8.032666	6	6	3	1	0
2023-H2	14592	5	3.426535	4	3	3	1	0
2024-H1	13475	9	6.679035	4	4	3	0	0
2024-H2	12370	6	4.850445	3	2	3	0	0
2025-H1	12384	19	15.34238	12	5	11	2	1

**Figure 1:**
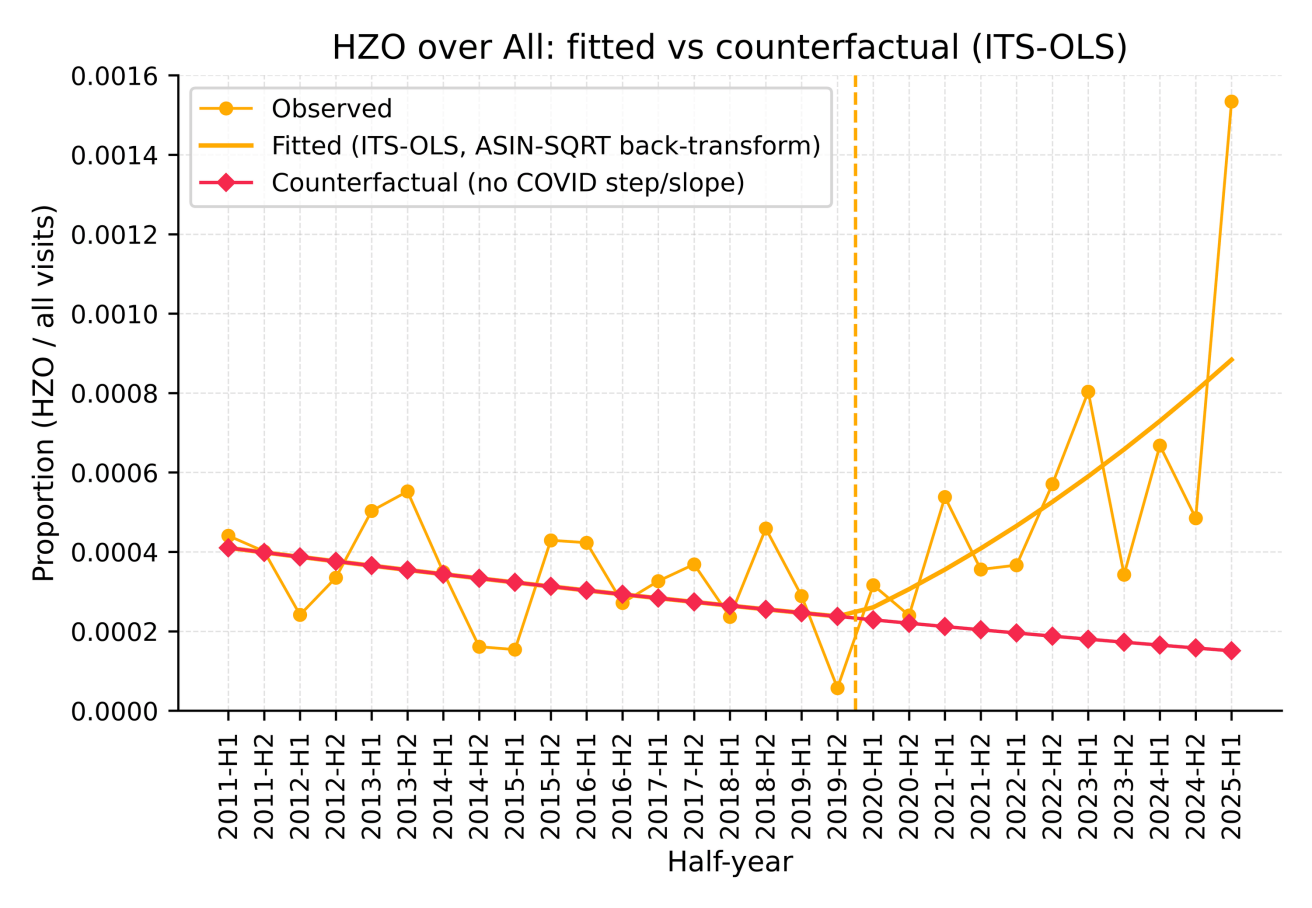
HZO proportion over time with segmented time series analysis (2011–2025). The proportion of ophthalmic herpes zoster (HZO) cases among all outpatients is plotted over time. Yellow data points and line indicate the observed proportion in each half-year. The orange solid line shows the fitted trend based on the ITS model, and the red solid line shows the counterfactual trend projected if the COVID-19 pandemic had not occurred. The vertical dashed line marks the start of the COVID-19 pandemic (first half of 2020). In the post-pandemic period, the fitted trend (orange) diverges upward from the counterfactual trend (red), indicating a significantly higher incidence than would be expected without the pandemic.

HZO relative frequency and ocular complication trends are summarized in Table 3. Prior to the intervention, the relative frequency of HZO was either gradually increasing or relatively flat. However, since the first half of 2020 (the start of the pandemic), the relative frequency of HZO showed a pronounced increasing trend (Figure 1). In the segmented regression analysis, the coefficient β3 representing the change in slope after the pandemic onset was significantly positive (β3 = 0.0016, 95% CI 0.0007 to 0.0025; Wald z = 3.60, p < 0.001), indicating that the rate of increase in the relative frequency of HZO became statistically significantly steeper following the start of the pandemic. In contrast, the level change at the intervention point (β2) was slight (a small drop/increase) and not statistically significant (β2 = -0.0006, 95% CI -0.0073 to 0.0061; Wald z = -0.19, p = 0.852), suggesting there was no immediate jump or drop in the relative frequency of HZO right after the pandemic began. Over the entire study period, the model-estimated cumulative excess number of HZO cases during the post-pandemic period was 55.2 (95% CI 17.4 to 84.7). In other words, about 55 more HZO patients were seen in our facility during the post-pandemic period than would be expected had the pandemic not occurred. It should be noted that this figure is a model-based estimation, not a directly observed count.

**Table 3:** Summary of segmented time series analysis (ITS) for HZO and ocular complications. Shown are the estimated immediate level change (β2) and trend change (β3) after the start of the COVID-19 pandemic. The model-estimated cumulative excess cases represent the differences between the fitted post-pandemic values and the counterfactual predicted values (assuming no pandemic) during the post-pandemic period. A statistically significant increase in the post-pandemic trend (slope) was observed for HZO (p < 0.001) and for uveitis (p = 0.024). OLS: ordinary least squares

Outcome	β2 estimate (95% CI)	Test statistic β2	P-value β2	β3 estimate (95% CI)	Test statistic β3	P-value β3	Cumulative excess cases (95% CI)
HZO (Total)	-0.0006 (-0.0073 to 0.0061)	z = -0.19 (segmented OLS)	0.852	0.0016 (0.0007 to 0.0025)	z = 3.60 (segmented OLS)	<0.001	55.2 (17.4 to 84.7)
Any ocular complication	-0.0020 (-0.0107 to 0.0068)	z = -0.44 (segmented OLS)	0.660	0.0009 (-0.0002 to 0.0021)	z = 1.58 (segmented OLS)	0.113	18.2 (-22.4 to 49.5)
Keratitis	-0.0021 (-0.0099 to 0.0056)	z = -0.54 (segmented OLS)	0.590	0.0005 (-0.0006 to 0.0015)	z = 0.85 (segmented OLS)	0.395	2.7 (-36.2 to 28.9)
Uveitis	0.0009 (-0.0074 to 0.0092)	z = 0.20 (segmented OLS)	0.839	0.0013 (0.0002 to 0.0024)	z = 2.25 (segmented OLS)	0.024	29.3 (3.4 to 47.1)
Ocular motility disorder	0.0018 (-0.0038 to 0.0074)	z = 0.64 (segmented OLS)	0.522	0.0002 (-0.0005 to 0.0010)	z = 0.65 (segmented OLS)	0.516	3.0 (-3.2 to 6.8)
Optic neuritis	0.0013 (-0.0021 to 0.0046)	z = 0.73 (segmented OLS)	0.462	0.0000 (-0.0004 to 0.0005)	z = 0.20 (segmented OLS)	0.838	0.4 (-1.0 to 1.3)

**Figure 2:**
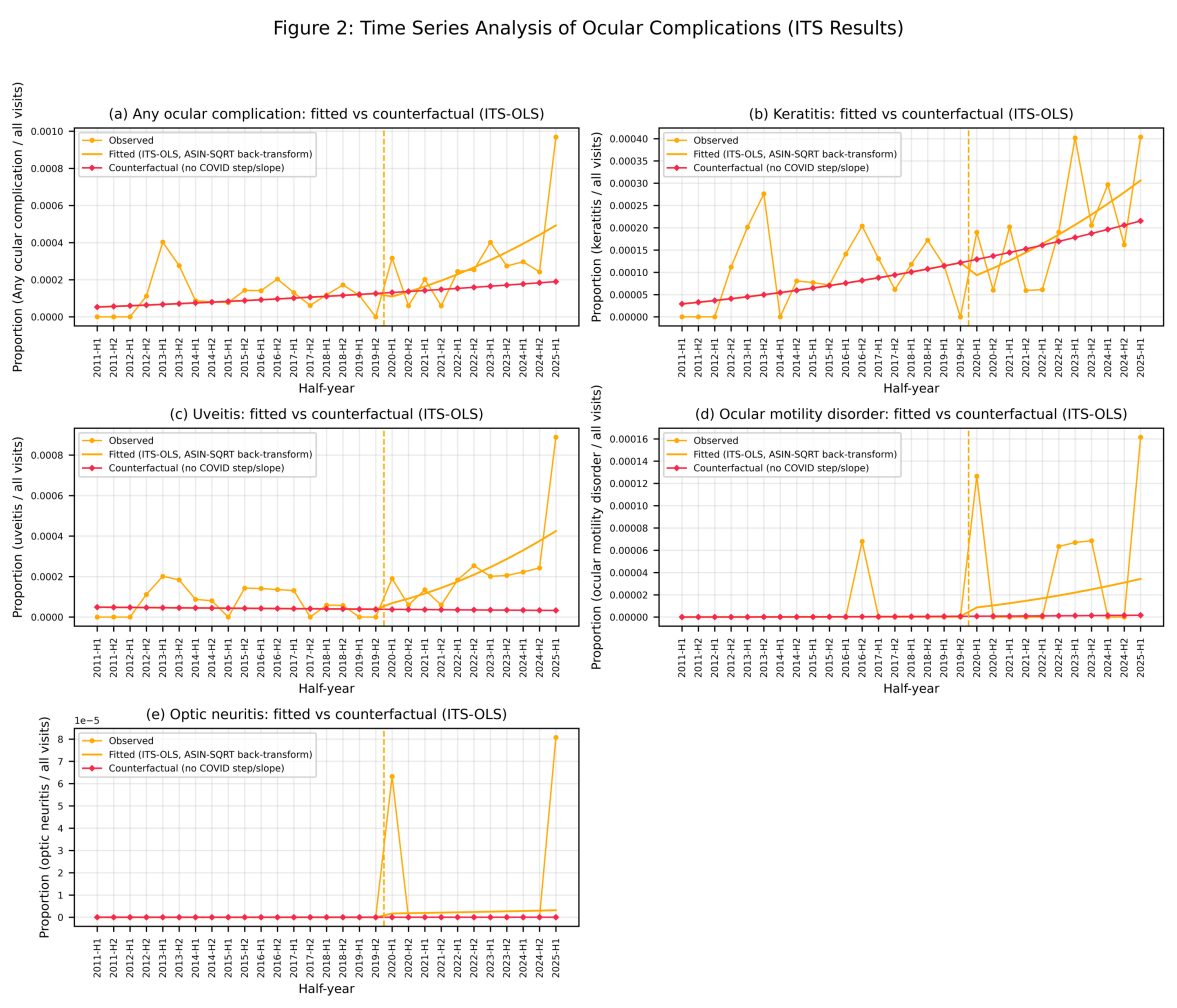
Trends in the relative frequency of ocular complications and ITS analysis results. Segmented time series analysis for each ocular complication: (a) Any ocular complication, (b) Keratitis, (c) Uveitis, (d) Ocular motility disorder, (e) Optic neuritis. The yellow line represents observed data; the orange line represents the fitted model; the red line represents the counterfactual scenario without the pandemic. Only in the case of uveitis (panel c) was there a statistically significant increase in trend and a notable number of excess cases post-pandemic. For the other complications, no significant deviation from the pre-pandemic trend was observed.

We applied the same ITS analysis to major ocular complications associated with HZO. For herpetic uveitis, similar to overall HZO, there was no immediate surge at the start of the pandemic; however, the long-term increasing trend significantly accelerated during the post-pandemic period (β3 = 0.0013, 95% CI 0.0002 to 0.0024; Wald z = 2.25, p = 0.024), resulting in a model-estimated cumulative excess of 29.3 cases (95% CI 3.4 to 47.1). This indicates a significant rise in the frequency of new-onset herpetic uveitis over time following the pandemic. In contrast, other complications - such as keratitis, ocular motility disorder, and optic neuritis - exhibited neither an immediate level change nor a statistically significant change in their long-term trends (see Figure 2).

When considering all ocular complications combined (“any ocular complication”), the trend change after the pandemic was not significant either (β3 = 0.0009, 95% CI -0.0002 to 0.0021; Wald z = 1.58, p = 0.113). However, the point estimate did show an upward tendency, suggesting that the lack of significance might be due to limited case numbers and hence insufficient statistical power. The proportion of patients with any ocular complication was higher in the post-pandemic period, but this difference did not reach statistical significance.

Model diagnostics did not identify meaningful residual autocorrelation in the primary segmented OLS models. For the main HZO model, the Durbin-Watson statistic was 2.10, and Ljung-Box tests were non-significant at lag 1 (Q=0.70, p=0.402) and lag 2 (Q=1.78, p=0.410). Similar non-significant Ljung-Box results were observed for the ocular complication models (Table 4). Residual augmented Dickey-Fuller tests rejected a unit-root pattern for all outcomes, supporting the adequacy of the deterministic segmented specification, although these tests should be interpreted cautiously given the limited number of time points.

**Table 4:** Residual diagnostics, stationarity checks, and seasonality assessment

Outcome	Durbin-Watson	Ljung-Box Q(1)	p Q(1)	Ljung-Box Q(2)	p Q(2)	Residual ADF statistic	p ADF	Season H2 coefficient	Season H2 z	p H2	Season-adjusted β3	Season-adjusted β3 z	p β3 season-adjusted
HZO	2.104188	0.703007	0.401775	1.781357	0.410377	-4.48072	0.000213	-0.00211	-1.32898	0.183856	0.001625	3.606882	0.000309898
Any ocular complication	1.625214	0.345459	0.556695	0.349721	0.839574	-3.58972	0.005961	-0.00265	-1.27554	0.202119	0.000918	1.561631	0.118375039
Keratitis	1.907043	0.010442	0.91861	0.266521	0.875237	-4.10554	0.000948	-0.001	-0.52418	0.600156	0.000442	0.821067	0.411608041
Uveitis	1.443771	1.104792	0.293217	1.212004	0.545528	-3.6681	0.004585	-0.00083	-0.40618	0.684608	0.001268	2.199126	0.027868932
Ocular motility disorder	1.948301	0.067261	0.795368	3.205026	0.20139	-4.91852	3.21E-05	-0.00025	-0.18382	0.854156	0.000246	0.631214	0.52790052
Optic neuritis	1.598664	0.013234	0.908415	0.068078	0.966534	-2.97353	0.037442	-0.00107	-1.34522	0.178555	3.70E-05	0.164019	0.869716169

No statistically significant half-year seasonality was detected. In season-adjusted models including an H2 indicator, the seasonal coefficient was not statistically significant for HZO (z = -1.33, p = 0.184) or for any ocular complication outcome. Importantly, the post-pandemic slope change for HZO remained significant after season adjustment (β3=0.0016, 95% CI 0.0007 to 0.0025; Wald z=3.61, p<0.001), and uveitis also remained significant (β3=0.0013, 95% CI 0.0001 to 0.0024; Wald z=2.20, p=0.028).

The sensitivity analyses generally supported the primary proportion-based findings. For HZO, the binomial-logit model and the Poisson rate model with a log outpatient-visit offset both showed a significant post-pandemic slope increase (OR/IRR per half-year slope change=1.171, 95% CI 1.078 to 1.272; p<0.001). In the absolute-count Poisson model without the outpatient-visit denominator, the HZO slope-change estimate remained positive but was not conventionally significant (IRR=1.074, 95% CI 0.991 to 1.165; p=0.081), suggesting that pandemic-related changes in outpatient utilization may have contributed to the magnitude of the proportion-based effect. For uveitis, the post-pandemic slope increase remained significant in the binomial-logit, Poisson rate, and absolute-count Poisson sensitivity models (Table 5).

**Table 5:** Sensitivity analyses for the principal outcomes GLM: Generalized linear model; OLS: Ordinary least squares

Outcome	Sensitivity model	Effect estimate (95% CI)	Test statistic	P-value
HZO	Primary arcsine-OLS proportion	β3=0.0016 (0.0007 to 0.0025)	Wald z=3.60	<0.001
HZO	Season-adjusted arcsine-OLS (H2 indicator)	β3=0.0016 (0.0007 to 0.0025)	Wald z=3.61	<0.001
HZO	Binomial-logit GLM	OR=1.171 (1.078 to 1.272)	Wald z=3.76	<0.001
HZO	Poisson rate GLM with log(total visits) offset	IRR=1.171 (1.078 to 1.272)	Wald z=3.76	<0.001
HZO	Absolute-count Poisson GLM (no denominator offset)	IRR=1.074 (0.991 to 1.165)	Wald z=1.74	0.081
Uveitis	Primary arcsine-OLS proportion	β3=0.0013 (0.0002 to 0.0024)	Wald z=2.25	0.024
Uveitis	Season-adjusted arcsine-OLS (H2 indicator)	β3=0.0013 (0.0001 to 0.0024)	Wald z=2.20	0.028
Uveitis	Binomial-logit GLM	OR=1.271 (1.098 to 1.471)	Wald z=3.22	0.001
Uveitis	Poisson rate GLM with log(total visits) offset	IRR=1.271 (1.098 to 1.471)	Wald z=3.22	0.001
Uveitis	Absolute-count Poisson GLM (no denominator offset)	IRR=1.162 (1.008 to 1.341)	Wald z=2.07	0.039

## Discussion

Through our ITS analysis, we found that although there was no significant immediate surge in the relative frequency of HZO right after the COVID-19 pandemic began (no level change), the subsequent rate of increase in the relative frequency (slope) became significantly steeper. In addition, among HZO-related ocular complications, uveitis in particular showed a similar significant increasing trend post-pandemic. These findings align with the report by Zong et al. that noted a sharp rise in herpetic uveitis diagnoses in patients 65 years and older since 2020 [9]. Although the increase in uveitis was statistically significant, it was based on relatively small absolute numbers. Therefore, this finding should be interpreted cautiously and confirmed in larger cohorts. Previous short-term studies yielded mixed observations - some reporting an apparent decrease in HZ/HZO due to reduced healthcare utilization, and others noting temporary increases possibly related to pandemic-associated stress. By using 15 years of data, our study provides novel evidence that the COVID-19 pandemic was associated with a persistent shift to a higher growth trend in the relative frequency of HZO, rather than just a short-lived fluctuation.

Our findings are broadly consistent with previous reports suggesting that HZO may have followed a distinct pattern over time. Kong et al. demonstrated a long-term increase in the proportion of HZO in a large retrospective cohort, suggesting that HZO may not necessarily parallel herpes zoster overall and may be influenced by distinct biological or clinical factors [10]. This interpretation is also compatible with our finding that the increase in HZO after the pandemic was not an abrupt level change, but rather a sustained acceleration in the relative frequency trend of HZO.

The reason for the post-pandemic increase in HZO cannot be determined from our data alone; however, several hypothetical explanations should be considered. First, it has been hypothesized that SARS-CoV-2 infection itself might potentially promote varicella-zoster virus (VZV) reactivation through dysregulation of cell-mediated immunity. Previous reviews have suggested that lymphopenia, T-cell dysfunction, and immune exhaustion after COVID-19 may impair immune surveillance against latent VZV and thereby facilitate viral reactivation [11]. Although we did not assess prior COVID-19 infection at the individual level, this mechanism remains biologically plausible.

Second, psychological and physical stress related to the pandemic could theoretically have contributed to the observed increase. Herpes zoster is more common in older adults and in individuals with impaired host immunity, and stress has long been considered a possible trigger for viral reactivation [6,12]. In our cohort, the mean age of patients with HZO increased significantly in the post-pandemic period, suggesting that aging of the affected population may have increased susceptibility to both VZV reactivation and more severe ocular inflammation. Thus, the post-pandemic increase in HZO may reflect not only pandemic-specific factors, but also their interaction with an increasingly older at-risk population.

Third, changes in healthcare-seeking behavior during the pandemic could theoretically have influenced the observed relative frequency pattern. During the early pandemic period, some patients with relatively mild symptoms may have avoided medical visits, whereas patients with facial rash, ocular symptoms, or more severe disease may have continued to seek care [13]. Such selective presentation could partly explain why HZO and uveitis appeared more prominent in the post-pandemic period.

The possible role of COVID-19 vaccination should be interpreted cautiously. Case reports and a recent systematic review have described HZO occurring after COVID-19 vaccination [14]. However, large epidemiologic studies have not consistently demonstrated an increased risk; for example, Akpandak et al. found no evidence of increased HZO risk after COVID-19 vaccination in a large U.S. claims database study [15]. Since vaccination status was not evaluated in this study, we cannot determine whether vaccination contributed to the observed increase, and no causal inference should be made on this point.

From a clinical standpoint, our findings highlight the importance of careful ophthalmologic evaluation in patients with HZO, particularly in older adults. Because HZO can lead to keratitis, uveitis, and other vision-threatening complications, the observed post-pandemic increase supports the importance of early diagnosis, prompt ophthalmologic referral, and preventive strategies such as herpes zoster vaccination in eligible populations [7,12].

Several limitations should be emphasized. First, the primary proportion metric used total ophthalmology outpatient visits as the denominator. This denominator is clinically useful because it reflects the burden of HZO and ocular complications within the outpatient service; however, it may also introduce bias during the COVID-19 pandemic. If total outpatient visits decreased during the early pandemic because of medical distancing, the same number of HZO cases could yield a higher proportion. To make this limitation transparent, we now report total outpatient visits and absolute HZO/complication counts by half-year and provide sensitivity analyses using absolute-count models. The HZO effect was strongest in proportion and rate models and weaker in the absolute-count model, so the findings should be interpreted as an increase in HZO burden relative to ophthalmology outpatient volume, not necessarily as a direct population-level incidence estimate.

Second, another limitation is the relatively small number of HZO cases and ocular complication events analyzed over the limited time points. Although the study covered a long observation period of 15 years, the total number of HZO cases was only 75 in the pre-pandemic period and 89 in the post-pandemic period, and the interrupted time series comprised only 29 half-year intervals. Consequently, some ocular complications were sparse, and even statistically significant slope changes should be interpreted cautiously, as the estimates may be affected by sparse events, influential time points, overfitting of the segmented regression model, or model instability for rare outcomes. This limitation applies not only to rare ocular complications but also to the main HZO trend analysis. Furthermore, although residual autocorrelation and half-year seasonality were not detected, these diagnostic tests have limited power in short time series. To address these concerns and reduce model-dependence, we utilized the arcsine square-root transformation as a pragmatic variance-stabilizing approach for small proportions, and performed residual diagnostics alongside sensitivity analyses using alternative model specifications (binomial-logit, Poisson rate, and absolute-count Poisson models). While consistency across the arcsine-OLS, binomial-logit, and Poisson rate models for HZO and uveitis supports the robustness of the main direction of the effect, the attenuation observed in some sensitivity models indicates that the results should be interpreted as hypothesis-generating rather than definitive evidence of a causal pandemic effect.

Third, important individual-level confounders were not available, including documented SARS-CoV-2 infection, COVID-19 vaccination status, comorbidities, immunosuppression, and antiviral or vaccine history. These factors could influence VZV reactivation risk, healthcare-seeking behavior, or disease severity. Therefore, the observed post-pandemic slope changes should be interpreted as associations with the pandemic period rather than causal effects of COVID-19 infection or vaccination. Multicenter studies with individual-level infection and vaccination data will be necessary to clarify mechanisms.

Fourth, diagnoses were based on routine clinical documentation rather than standardized prospective assessment or systematic PCR confirmation, thus misclassification bias is possible. Diagnostic practices may also have changed over the 15-year study period.

## Conclusions

In conclusion, our interrupted time series analysis suggests that the COVID-19 pandemic was associated with a sustained acceleration in the outpatient visit-based frequency trend of HZO, with a parallel increase in herpetic uveitis. Although the precise reason for this increase remains unclear, it is likely multifactorial and may involve immune dysregulation after SARS-CoV-2 infection, pandemic-related stress, aging of the affected population, and changes in healthcare-seeking behavior. Further multicenter studies incorporating individual-level infection history, vaccination data, and standardized ophthalmic evaluation will be needed to clarify the mechanisms underlying this post-pandemic increase in HZO.
